# Human Biomonitoring of Selected Hazardous Compounds in Portugal: Part II—Lessons Learned on Mycotoxins †

**DOI:** 10.3390/molecules27010130

**Published:** 2021-12-27

**Authors:** Angelina Pena, Sofia Duarte, André M. P. T. Pereira, Liliana J. G. Silva, Célia S. M. Laranjeiro, Marta Oliveira, Celeste Lino, Simone Morais

**Affiliations:** 1 LAQV, REQUIMTE, Laboratory of Bromatology and Pharmacognosy, Faculty of Pharmacy, University of Coimbra, 3000-548 Coimbra, Portugal; apena@ci.uc.pt (A.P.); andrepereira@ff.uc.pt (A.M.P.T.P.); ljgsilva@hotmail.com (L.J.G.S.); celialaranjeiro@gmail.com (C.S.M.L.); clino@ff.uc.pt (C.L.); 2 Centro de Investigação Vasco da Gama, Departamento de Ciências Veterinárias, Escola Universitária Vasco da Gama, Av. José R. Sousa Fernandes, Campus Universitário, 3020-210 Coimbra, Portugal; 3 LAQV, REQUIMTE, Instituto Superior de Engenharia, Instituto Politécnico do Porto, R. Dr. António Bernardino de Almeida 431, 4200-072 Porto, Portugal; Marta.Oliveira@graq.isep.ipp.pt (M.O.); sbm@isep.ipp.pt (S.M.)

**Keywords:** biomarkers of exposure, mycotoxins, health risks

## Abstract

Human biomonitoring (HBM) data provide information on total exposure regardless of the route and sources of exposure. HBM studies have been applied to quantify human exposure to contaminants and environmental/occupational pollutants by means of determining the parent compounds, their metabolites, or even their reaction products in biological matrices. HBM studies performed among the Portuguese population are dispersed and limited. Thus, to overcome this knowledge gap, this work reviews the published Portuguese HBM information concerning mycotoxins detected in the urine, serum, milk, hair, and nails of different groups of the Portuguese population. This integrative approach to the available HBM data allows us to analyze the main determinants and patterns of exposure of the Portuguese population to the selected hazardous compounds, as well as to assess the potential health risks. We also aimed to identify the main difficulties and challenges of HBM through the analysis of the enrolled studies. Ultimately, this study aims to support national and European policies in promoting human health by summarizing the most important outcomes and lessons learned through the HBM studies carried out in Portugal.

## 1. Introduction

In everyday life, humans are exposed to a broad range of hazardous substances and their mixtures, which are present in air, soil, water, and food. It is of the utmost importance to gather scientific evidence on these in order to provide early protection for human health, since some of these chemicals cause deleterious effects, and prolonged human exposure, even at low doses, can be related with chronic diseases and cancer [1]. Different approaches can be followed, namely, the assessment of environmental/occupational levels of hazardous pollutants and food contaminants, and/or the determination of the cumulative chemical burden through human biomonitoring (HBM) actions [2]. HBM represents an adequate tool to assess human exposure to hazardous substances and/or the associated health risks through the measurement of chemicals and their metabolites or reaction products in biological matrices (e.g., blood, urine, breastmilk, saliva, etc.) [3]. HBM studies allow the determination of total exposure to mixtures of contaminants/pollutants with growing concern for human health risk assessments, regardless of the route of exposure (inhalation, ingestion, or dermal uptake) and taking into account personal characteristics and individual lifestyles [4,5]. HBM can help to find: (1) new emerging chemical exposures, as well as new trends and variations in such exposure; (2) populations or groups that are more vulnerable or have higher exposures; (3) the patterns of exposure not only among the general population but also among specific population groups. The use of HBM studies can help to clarify the association between environmental/occupational exposure with personal internal exposure and early health risks; however, no causal correlation can be established, either in terms of the identification of the sources or the route of exposure. When performed over time, HBM studies allow the assessment of exposure trends, and comparison of the data obtained with the available reference guidelines and/or with the values obtained for control groups helps to assess the health risks for exposed individuals and to conduct corrective actions if necessary [6]. Moreover, data generated with HBM studies should be communicated to health professionals, regulators and policy makers, as they are of great relevance to health risk management, in particular through the implementation of measures to prevent exposure and to mitigate the identified risks [7]. HBM has been seldom performed simultaneously with the collection of environmental exposure data [8,9,10]. Additionally, the majority of HBM studies only consider exposure to one or a few chemicals at a time [11]. Still, the HBM4EU initiative, a European project with 30 participating countries, including Portugal, and with the support of the European Environment Agency (https://www.hbm4eu.eu/, accessed on 22 December 2021), is coordinating and advancing HBM across Europe. It has defined a list of priority hazardous substances including, but not limited to, emerging substances, flame retardants, phthalates, polycyclic aromatic hydrocarbons (PAHs), pesticides, benzophenones, mycotoxins, and some heavy metals and metalloids [12]. Several HBM studies have been performed among the Portuguese population; however, the available information remains dispersed. Thus, the present work aims to bring together the information retrieved from HBM studies related with the Portuguese population’s exposure to mycotoxins over the last 15 years. A critical review of the available information is performed, taking into consideration the existent national and international guidelines. Moreover, by integrating the main challenges and lessons learned through Portuguese HBM studies, the main potential health risks are also reviewed, contributing support for safety, health, and environment policies in Europe.

## 2. Methodology

The available scientific literature was searched using the Thomson Reuters ISI Web of Knowledge, PubMed, Science Direct, and Google Scholar databases. Combinations of at least two of the following keywords were used: “Portugal”, “Portuguese”, “human biomonitoring”, “biomarkers of exposure”, and “mycotoxins”. All the HBM studies assessing exposures to mycotoxins within the Portuguese population were selected.

The inclusion criteria for the selected studies were the determination of mycotoxins and/or their biomarkers of exposure in biological fluids and having full access to the study; studies not reporting original data or surveying populations not including Portuguese subjects were excluded. Overall, the literature search identified a total of 18 HBM studies published between 2006 and 2020.

## 3. Mycotoxins

Mycotoxins are secondary metabolites of low molecular weight, produced before and after harvest, by several species of filamentous fungi [13]. The presence of these chemically diverse substances in feed and food crops cannot be completely avoided under current agricultural practices [14]. Thus, the main route of exposure to mycotoxins, both for humans and animals, is through the ingestion of contaminated food, although exposure through dermal contact or inhalation may occur, notably in the context of occupational exposure [15,16]. Among the numerous pathophysiological effects of mycotoxins, the primary concern relates to chronic effects resulting from low levels of exposure. However, proving mycotoxin exposure and establishing a diagnosis of mycotoxicosis are hindered not only by their general insidious nature but also by the different factors influencing the pathogenesis of the disease [17]. Mycotoxins are considered highly relevant agricultural contaminants since several are classified by the IARC as known and/or potential human carcinogens [18]. Mycotoxins feature a marked resistance to most technological food processing techniques, along with a global occurrence and a broad variety of foodstuffs that are susceptible to contamination. In the specific case of Portugal, the climate promotes the growth of various mycotoxigenic molds and hence contributes to a higher risk of mycotoxin production. Furthermore, as part of the traditional Mediterranean diet, the Portuguese diet is characterized by a food pattern with a high consumption of plant foods, such as poorly refined cereals, and dried nuts. It is also noteworthy that a significant part of the food consumed in the country is imported from countries outside the EU without any maximum levels or regulations, nor any monitoring of the presence of mycotoxin contamination [17].

### 3.1. Aflatoxins

Concerning human toxicity, aflatoxins (AFs) represent the group of mycotoxins of greatest concern, as they are considered one of the most potent natural carcinogens [18]. AFs are produced by *Aspergillus* species, namely, *A. flavus* and *A. parasiticus.* These are widely found in several agricultural crops, including cereals such as corn, nuts such as peanuts and almonds, and spices [19]. The presence of AFs in foods has a worldwide distribution, predominantly in regions with a tropical and subtropical climate. Aflatoxin B1 (AFB1) exerts its hepatotoxic, teratogenic, mutagenic, and carcinogenic effects after biotransformation into the reactive compound AFB1-epoxide by means of cytochrome P450 (CYP) enzymes. This epoxide can react with nucleic acids and proteins and cause mutation in codon 249 of the tumor suppressor gene p53 [20]. CYP enzymes also metabolize AFB1 into the aflatoxin M1 (AFM1), the main metabolite. This metabolite can thus be used as a biomarker of exposure to AFB1 [21,22,23]. AFB1 and AFM1 are IARC Group 1 carcinogens, with the first featuring at least 10 times higher carcinogenic potency [24]. The acute toxicity is comparable between both mycotoxins [23,25]. Furthermore, AFs present acknowledged immunosuppressive effects [19]. In children, exposure to aflatoxins may also be associated with growth retardation, malnutrition, and neurological impairment [26]. Breastfed babies are recognized as particularly vulnerable to AFM1 toxicity due to their high metabolism and consumption per kilogram of body weight, their restricted diet, and their low detoxification capability [27].

In the first AFM1 breastmilk biomonitoring study conducted in Portugal [28] (Table 1), roughly 33% of the analyzed samples contained detectable levels of AFM1 (7.4 ± 1.9 ng L^−1^), with values reaching 10.6 ng L^−1^. The determined incidence rate was comparably higher than in HBM studies performed in Brazil [22], Iran [21,29], and Turkey [30]. The average and maximum levels were comparable with studies previously conducted in Cyprus [31] and Lebanon [32]. However, the maximum level determined among the Portuguese population was nearly 10 times below the maximum levels registered in studies conducted in Jordan [33] and Egypt [34]. It was also noteworthy that none of the analyzed Portuguese breastmilk samples surpassed the maximum limit set in the EU for commercial infant and follow-on formulae (25 ng L^−1^ [35]). Variations in the analytical performance of the methods applied in each of the studies may justify the differences between the incidences and levels reported in the HBM surveys [28].

In the Portuguese survey performed by Bogalho et al. [28], it was possible to identify some determinants of exposure, given that all the participating mothers completed a questionnaire covering lactation, socio-demographic, and food consumption (7-day recall period) information. AFM1 contamination of breastmilk was associated with statistical significance to the mother’s lower level of education and higher consumption of chocolate and rice. Contamination was also associated with the early stage of lactation and the summer season. Although without statistical significance, several trends of food consumption were further identified, namely, the consumption of yogurts, coffee, cereals, and cereal-derived foods, such as cookies. The AFM1 estimated daily intake (EDI) in the Portuguese study [28] demonstrated that both the youngest (with less than 7 kg; 1.06 ng kg^−1^ b.w./day) and the oldest (heavier than 7 kg; 0.86 ng kg^−1^ b.w./day) subjects exceeded the proposed tolerable daily intake (TDI = 0.2 ng kg^−1^ b.w.) [36]. These findings were of concern given the mentioned features that render breastfed infants more vulnerable to AFM1 exposure. This is also of concern because above the level of 1.0 ng kg^−1^ b.w./day, there is a risk of hepatic cancer [37].

Although exposure is mainly of a foodborne nature, occupational exposure can further contribute to the total exposure. Previously, occupational exposure to AFB1 was assessed through the biomonitoring of blood of Portuguese workers from waste management [38], and from swine [39] and poultry [40,41] farms and slaughterhouses [38]. Overall, higher levels were detected among these workers, in comparison with the respective control groups comprising subjects without any type of agricultural activity, in which AFB1 was not detected (Table 1). Despite AFB1in blood is not considered a validated biomarker of exposure to dietary intake of aflatoxins, it was considered in this work because it was measured in these cited studies regarding a potential occupational exposure. In a recent HBM study conducted among 25 swine production workers, urinary AFM1 was the second most frequent mycotoxin encountered in a total of 42 mycotoxins considered (16%), after deoxynivalenol-glucuronic acid conjugate (52%) [42].

**Table 1:** The occurrence and levels (ng L^−1^) of aflatoxins (AFM1 and AFB1) in different biological samples collected among the Portuguese population.

Biomarker	Matrix	Sample	Incidence (%)	Range	Average ± SD	Reference
**AFM1**	Urine	Swine farm workers	4/25 (16%)	(n.d.–5400)	4900	[42]
**AFM1**	Breastmilk	Breastfeeding mothers	22/67 (32.8%)	(n.d.–10.6)	7.4 ± 1.9	[28]
**AFB1**	Blood serum	Waste management workers Control group	41/41 (100%) 0/30	(2500–25,900) n.d.	9900 ± 5400 n.d.	[38]
**AFB1**	Blood serum	Poultry slaughterhouse workers Control group	14/30 (47%) 0/30	(1060–4030) n.d.	1730 n.d.	[43]
**AFB1**	Blood serum	Poultry farm workers Control group	18/31 (59%) 0/30	(n.d.–4230) n.d.	2000 ± 980 n.d.	[41]

### 3.2. Ochratoxins

The most important and most frequently occurring members of the ochratoxins family is ochratoxin A (OTA). OTA has been reported as a widespread food contaminant, principally in cereals and their derivatives (e.g., bread, flour, and breakfast cereals) [44]. OTA is categorized as a possible human carcinogen by IARC (group 2B) and numerous toxic effects were described in animal models, namely, hepatotoxicity, neurotoxicity, teratogenicity, and immunotoxicity [45]. Regardless of the source of exposure and the animal species considered, OTA exerts primarily nephrotoxic effects [46] and an epidemiological association between OTA food exposure and biomarkers of exposure has already been demonstrated in the etiology of endemic nephropathy in the Balkan region [47], whereas chronic interstitial nephropathy has been reported in northern Africa countries, such as in Egypt [48] and Tunisia [46,49,50]. The toxicokinetics of OTA determine not only its toxicity, but also the features of biomonitoring. The unfavourable OTA elimination kinetics in humans contribute to its fairly long serum half-life (T_1/2_; 35 days), which is particularly useful in HBM studies [51,52].

Taking into account the exposure assessments through OTA blood biomarkers, the population of central Portugal has been the most frequently studied (Table 2). OTA blood exposure biomarkers were initially studied in individuals under hemodialysis, living in the cities of Coimbra and Aveiro [53]. Overall, hemodialysis patients living in Coimbra presented slightly higher levels of serum OTA than in healthy controls (500 ± 290 vs. 420 ± 180 ng L^−1^), which can be justified by the positive effect of the dialysis treatment (Table 2). Furthermore, for subjects living in the city of Aveiro, men presented higher levels than women (520 ± 240 vs. 440 ± 180 ng L^−1^). In a study by Lino et al. [54] all the 104 healthy residents from Coimbra (urban) and two nearby villages (rural) presented detectable levels of OTA. No association was found between OTA levels with the gender of participants (men vs. women) or their residence (rural vs. urban). Nevertheless, it was reported that men featured higher mean levels than women (460–1010 vs. 380–600 ng L^−1^). In addition, populations from the two rural villages presented higher serum values (780 ± 530 and 440 ± 310 ng L^−1^) than those living in the Coimbra urban area (420 ± 180 ng L^−1^). The authors justified such differences based on differences in climate conditions and dietary habits.

Even though OTA exposure occurs mainly through food consumption, occupational exposure has also been demonstrated through blood HBM studies. Viegas et al. [55] demonstrated a high exposure to OTA, concerning both incidence and levels, in workers from a waste sorting plant. However, the authors simultaneously determined, in the same blood samples, a high occurrence of 2′R-ochratoxin A, an OTA degradation product formed only during coffee roasting and thus related to coffee consumption. Enniatin B, a *Fusarium* mycotoxin also surveyed in the same study, was found in the serum of all workers from the waste sorting plant, although at much lower levels (10 to 150 ng L^−1^).

OTA biomarkers in urine are considered a promising alternative in exposure assessments. Indeed, despite the higher OTA serum levels, OTA in urine has demonstrated improved correlations with food consumption. Nevertheless, the small levels of the mycotoxin in urine require the adoption of analytical methodologies with higher sensitivity [52]. Another disadvantage reported by Duarte et al. [56] is the high intra-individual variation of the levels of OTA in urine, confirming OTA as a short-term exposure biomarker. Thus, just like serum OTA levels, urine OTA levels are more useful in characterizing the exposure of a (sub)population, rather than at the individual level.

One study [56] reported a nationwide Portuguese two-year survey enrolling 472 participants (Table 2). The urine biomonitoring in four regions (Porto, Coimbra, Lisboa, and Alentejo) showed a high incidence (86%) although at low average levels (19 ng L^−1^). Considering previous studies from other countries, as reviewed by Malir et al. [46] the incidence was among the highest reported, whereas the mean levels were the lowest. The population from the Alentejo region was the most exposed, as revealed by the highest incidence of contamination and mean levels. In addition to different climate conditions, the authors identified different food consumption patterns and socioeconomic levels as potential determinants of exposure. Considering all the four studied regions, no significant difference was found between years and seasons, but samples collected in winter featured higher contamination levels with a difference close to significance (*p* = 0.0623). It could thus be reasonable to consider that besides climate conditions, a possible seasonal difference in eating habits can cause variations in food intake throughout the year. The major contribution of transversal consumption throughout the population, regardless of socio-demographic features, in terms of the OTA exposure of a staple food, could explain the lack of correlation with potential socio-demographic determinants studied [56]. Two previous studies [57,58] analyzing urine-collected winter samples from healthy inhabitants of Coimbra, presented lower frequencies of contamination (Table 2). It is worth mentioning that the urine collection in the study reporting the lowest incidence (43% [58]) was carried out during the driest winter registered in 80 years.

More recently, a multi-mycotoxin study in Portugal evaluated 24-h and first-morning urine paired samples from 94 participants enrolled within the scope of the National Food, Nutrition, and Physical Activity Survey of the Portuguese General Population (2015–2016) [59]. The analysis revealed 11 and 12 out of the 37 mycotoxin biomarkers of exposure in 24-h urine and first-morning urine samples, respectively. OTA was detected in 27% of first-morning urine samples, respectively, confirming the exposure of the Portuguese population to this mycotoxin. The concentrations determined in first-morning urine samples ranged between 7–610 ng L^−1^. OTα was not detected. It should be mentioned that the average OTA levels determined in this study were 3- to 10-fold lower than those reported in other European countries, which can be justified by differences in the analytical methods applied [59].

Considering this reported widespread occurrence, a recent study [60] analyzed OTA exposure in children between 2 and 13 years old. Although considered a more susceptible population, infant exposure to this mycotoxin had only been surveyed in three previous studies in Cameroon [61], Sierra Leone [62] and Belgium [63]. The first survey that analyzed OTA in urine samples from Portuguese infants showed widespread OTA exposure. Indeed, in samples from the 85 healthy children enrolled, the majority (92.94%) were found to be positive, with up to 52 ng L^−1^ (114.45 ng g^−1^ of creatinine). Furthermore, taking into account the mean OTA levels determined, the risk assessed ranged from 10% to 194%, and were thus of concern [60].

Adult occupational exposure to OTA also showed widespread contamination (80%) through analyzed urine samples from workers from swine farms, although with only a single sample higher than the limit of quantification [42]. Control groups also showed widespread urine contamination (68%), which suggests that inhalation in the context of occupational exposure could additionally contribute, although less significantly, to exposure to OTA. Exposure assessments in another occupational setting (a fresh dough company) revealed that OTA was the second most prevalent mycotoxin (after deoxynivalenol glucuronide), although the control group presented a higher frequency of contamination. In both working and control groups, OTA was below the limit of quantification [64].

**Table 2:** The occurrence and levels of OTA (ng L^−1^) in different biological samples collected among the Portuguese population.

Biomarker	Matrix	Sample	Incidence (%)	Range	Average ± SD	Reference
**OTA**	Urine	Children (2–13 years old)	79/85 (92.94%)	(n.d.–52)	20 ± 13	[60]
**OTA**	Urine	Swine farm workers Control group	20/25 (80%) 13/19 (68%)	(n.d.–100) <LOQ	100 <LOQ	[42]
**OTA**	Urine	Fresh dough company workers Control group	10/21 (48%) 13/19 (68%)	<LOQ <LOQ	<LOQ <LOQ	[64]
**OTA**	Blood serum	Waste management workers Control group	42/42 (100%)	(441–6047)	1007	[55]
**R-OTA**	Blood serum	Waste management workers Control group	34/42 (81%)	(n.d.–627)	334	
**OTA**	Urine	All participants	408/472 (86.4%)	(n.d.–122)	19 ± 14	[56]
		Porto	90/111 (81.1%)	(n.d.–62)	17 ± 10	
		Coimbra	77/94 (81.9%)	(n.d.–69)	16 ± 11	
		Lisboa	127/150 (85.3%)	(n.d.–94)	19 ± 14	
		Alentejo	113/117 (96.6%)	(n.d.–122)	23 ± 16	
**OTA**	Blood serum	General adult population (Coimbra)	104/104 (100%):	(190–960)	420 ± 180	[54]
**OTA**	Urine	General adult population	13/30 (43.3%)	(n.d.–208)	19 ± 41	[58]
**OTA**	Blood serum	Hemodialyzed patients from: -Coimbra Aveiro	50/50 (100%) 45/45 (100%)	120–1520 150–1030	500 ± 290 490 ± 220	[53]
**OTA**	Urine	General adult population	42/60 (70%)	(n.d.–105)	38	[57]
**OTA**	Urine	General adult population	27%	7–610		[59]

### 3.3. Fumonisins

Fumonisins (FBs), mycotoxins with different structurally related analogues, are predominantly produced by *Fusarium verticillioides* and *F. proliferatum,* which are present in maize and its derivatives [65,66]. The use of contemporary agricultural practices, the existence of regulated food processes, marketing systems, and legislated contamination levels have significantly decreased the human exposure to mycotoxins. Structurally similar to sphinganine (Sa) and sphingosine (So), fumonisins inhibit ceramide synthase and block the biosynthesis of complex sphingolipids, causing several biological effects in animals and humans [67]. In the Transkei region, in South Africa and China, fumonisin B1 (FB1), the most prevalent and toxic fumonisin [68,69], was epidemiologically related to human esophageal cancer [66], whereas in South Texas, USA, it was associated with neural tube defects [70]. Therefore, FB1 was classified by the IARC as possibly carcinogenic to humans, Group 2B [71].

However, to assess the impact of FBs on human health, it is crucial to evaluate exposure by estimating the EDI through food consumption or by determining biomarkers that reveal the total exposure, overcoming issues such as differences in food contamination and consumption, diet habits, food preparation practices, as well drawbacks in terms of sampling representativeness and the accurate assessment of these parameters [72]. Given the non-existence of quantifiable metabolites, FB_1_ has been recommended as a biomarker. Studies on toxicokinetics with labeled and unlabeled FBs have demonstrated that a portion of the amount ingested is excreted via urine [73,74] and consequently urine, instead of plasma or feces, can be considered a good indicator to monitor human exposure [61,72,73,75,76,77,78].

An HBM study assessed the urinary levels of FBs in both rural and urban populations from the central zone of Portugal [77]. Those authors found that none of the 68 subjects presented detectable levels of FB1 or fumonisin B2 (FB2), which can be explained by their low bioavailability given the reduced exposure levels and rapid elimination from the body [72]. In addition, only up to 1% of the ingested FB1 is excreted through urine [74]. Recently, the above-mentioned multi-mycotoxin study reported that FB1 was found in 7% and 3% of 24-h urine and first-morning urine samples, respectively. The biomarkers FB2, fumonisin B3 (FB3), and the hydrolysed metabolite HFB1 were not detected in any of the analyzed samples [59].

Other studies have also recommended the use of FB1 and FB2 as biomarkers of exposure to FBs, principally in populations with short-term exposures and under high degrees of exposure [72,74,75,79]. HBM studies performed in Italy and Sweden detected the presence of FBs in human urine [80,81]. A multi-biomarker analytical methodology, applied to evaluate the prevalence and levels of FB biomarkers in the urine samples of 52 volunteers inhabiting the Apulia region in Southern Italy, showed that 56% of the study population presented FB1 [80]. Although maize and its derivatives do not belong to the typical Italian diet, they are usually consumed as chips, polenta, popcorn, beer, cornflakes, snacks, muesli, and mixed cereals. The mean concentrations of FB1 were 0.055 μg L^−1^, which represented an estimated human exposure that was lower than the TDI established for these mycotoxins [80]. Moreover, Gong et al. [76] and Westhuizen et al. [74] could positively correlate the consumption of tortillas and maize with urinary FB1 concentrations in Mexican and South African populations, respectively. However, there are HBM studies that were not able to detect FBs in the urine of German, Belgian, or Spanish subjects.

Extensive research on the biomarkers of FBs has been carried out based on their mode of action, specifically the inhibition of the biosynthesis of de novo sphingolipids. Ceramide synthase inhibition causes an elevation in the Sa concentration and, subsequently, an increase in the Sa-to-So ratio in various animal species and in humans [72]. In Portugal, the analysis of 68 human urine samples obtained from participants living in the Central zone of the country, namely, a rural and an urban area, showed that the Sa/So ratio was 0.43 ± 0.22 and 0.42 ± 0.17, respectively; no significant differences were found between populations [82]. Moreover, a prior study performed in this region revealed that these populations, even the rural one, were certainly under low exposure levels [82]. Data retrieved from Portuguese HBM studies comply with data found in the literature for French [83] and Italian [80,84] populations. Castegnaro et al. [83] investigated urine provided by 14 female and seven male healthy French participants, and verified normal values of the Sa/So ratio.

Nonetheless, a study in China [85] advocated that human sphingolipid metabolism can be influenced by the intake of FB1, and that the Sa/So ratio in urine may be helpful for assessing high FB1 exposure, claiming that males are more susceptible to FB1 inhibition of sphingolipid metabolism than females. In 2001, the potential role of FBs in endemic nephropathy, a chronic renal disease, was studied in Brodska Posavina, Croatia. The Sa/So ratio was evaluated in healthy participants and in patients from this endemic region. The results, both in urine and in serum, revealed sphingolipid metabolism damage, possibly caused by FBs or fumonisin-like mycotoxins. Since statistically significant differences were verified when comparing them to the participants not affected by endemic nephropathy, impairment in sphingolipid metabolism might be regarded as an initial sign of this disease [86].

Concentrations of FBs have also been determined in other human biological samples such as in serum [83,86] and plasma [84]. The average Sa/So ratio in the serum of nine healthy female participants from France was 0.43 (0.18–0.78), whereas in nine male participants it was 0.31 (0.11–0.57). In South Africa, in 13 female participants the ratio was 0.22 (0.09–0.44), and in patients with esophageal cancer it was 0.23 (range 0.16–0.36). Therefore, despite the small number of cancer patients (*n* = 4), no statistical difference was observed in the Sa/So ratio compared with the control group of esophageal cancer patients [83]; these results are also in accordance with those obtained in several other studies.

Notwithstanding the analytical progress made in the determination of Sa and So, some questions remain to be addressed. The ratio must be evaluated individually, along with FB exposure, and may only be helpful in highly exposed populations, with levels close to or above the established TDI.

### 3.4. Others

Deoxynivalenol (DON) is a tricothecene produced by *F. graminearum* and *F. culmorum.* Unmetabolized DON, along with its glucuronide conjugate, is among the major compounds found in human urine. However, some reports have also shown the presence of the metabolite deepoxy-deoxynivalenol (DOM-1) in human urine [87,88]. In Portugal, the natural occurrence of DON and its metabolites in human urine samples from the north zone of Portugal was preliminary evaluated in 2012 in 13 volunteers of both female and male sexes [87]. Free DON and total DON (free and conjugated) were detected in 15% and 69% of the samples, respectively. Free DON was found at the levels of 1800 and 8800 ng L^−1^, whereas total DON levels ranged from 1900 to 26,200 ng L^−1^ with a mean of 16.3 ng L^−1^. DON metabolites, DOM-1, 3-acetyldeoxynivalenol (3-AcDON) and 15-acetyldeoxynivalenol (15-AcDON) were not detected in any of the analyzed samples. These results were in agreement with those obtained by other researchers [88,89].

A recent Portuguese multi-mycotoxin study also reported the exposure of the Portuguese population to DON, zearalenone (ZEN), alternariol, and citrinin (CIT) [58]. DON and its metabolites (DOM-1, 3-AcDON, and 15-AcDON) were most frequently found in 24-h urine samples, at 63%, 41%, 44%, and 52%, respectively. Considering DON and its metabolites, 78% of participants were exposed to DON. The median concentration levels reported were of 2210, 240, 330, and 173 ng L^−1^ for DON, DOM-1, 3-AcDON, and 15-AcDON, respectively. In first-morning urine samples, DON and metabolites were the second most commonly detected biomarkers (30%, 32%, 11%, 24%, and 39%, respectively), confirming the results obtained for the 24-h urine samples [59].

Zearalenone (ZEN), a metabolite primarily associated with several Fusarium species is a mycoestrogen, along with its alcohol metabolites, α-zearalenol (α-ZEN) and β-zearalenol (β-ZEN). This non-steroidal estrogenic toxin was categorized into Group 3 (not classifiable as to its carcinogenicity to humans) by the IARC [90]. In the above-cited study, ZEN was the second most frequently detected mycotoxin with 48% of 24-h urine samples found to be positive. In first-morning urine samples, ZEN was the most frequent detected mycotoxin (57%). Regarding the metabolites, its glucuronide conjugate, ZEN-14-GlcA, was detected in the same proportion for 24-h urine and first-morning urine samples, and α-ZEN was only detected in 5% of first-morning urine samples. The median concentration levels reported for 24-h samples were 170 ng L^−1^ for both ZEN and ZEN-14-GlcA, whereas for first-morning samples, levels of 1300, 150, and 2700 ng L^−1^ for ZEN, ZEN-14-GlcA, and α-ZEL, respectively, were found [59].

CIT is a polyketide mycotoxin produced by fungi belonging to the genera *Penicillium*, *Aspergillus*, and *Monascus* [91]. Exposure to CIT is of toxicological interest since it disturbs kidney function in several species, specifically in the renal tubules. CIT induced micronuclei in human-derived liver cells (HepG2) at levels equal to or greater than 10 μM and decreased in a dose-dependent manner the percentage of binucleated cells [91]. The Portuguese exposure to CIT was found to be low since it was detected in only 2% of both types of urine samples in median levels of 850 and 750 ng L^−1^, for 24-h and first-morning urine samples, respectively. Surprisingly, it was verified that samples that were positive for CIT were negative for OTA [59].

Alternariol (AOH), a *Fusarium* alternaria toxin, is regarded as an emerging toxin that also presents estrogenic activity and is regarded as a potential endocrine disruptor [92]. AOH was also recently identified in Portuguese urine samples, confirming the human exposure to this mycotoxin [59]. The presence of AOH in 24-h urine samples correlated well with first-morning urine samples, with median values of 280 and 210 ng L^−1^. AOH was identified for the first time in urine samples from a European country [59].

## 4. Final Remarks

HBM has contributed to the availability of data related to exposure to mycotoxins in the Portuguese population. The identified exposure determinants could be the starting point for further studies and health promotion policies and programs, particularly in population groups that were found to be more frequently associated with higher exposure to mycotoxins.

Among the several mycotoxins that were included in the studies, OTA and AFs were the predominant ones. A recent HBM study demonstrated infant exposure to OTA and AFs [60]. Indeed, some breastfed infants presented exposure levels to AFs that were five times greater than the TDI value proposed in [36] (0.2 ng kg^−1^ b.w.), and the mother’s consumption of chocolate and rice, a lower level of education, and the period when the samples were collected (summer and at the beginning of lactation) were potential determinants of exposure [28]. Serum concentrations of OTA were increased in rural populations from the central region of Portugal [54]. Furthermore, the available evidence demonstrated a high exposure to urinary biomarker in residents from Alentejo region [56]. Although to a lesser extent, the contribution of occupational exposure to mycotoxins was also demonstrated in different working settings. Despite the Portuguese participation in the European Human Biomonitoring Program HBM4EU, the present study revealed the limited nature of the existing information regarding the evaluation of Portuguese exposure to the selected hazardous substances. The difficulty in mobilizing a representative sample (by gender, age, region, and informed agreement) to study a wide range of health indicators and obtain more robust results was also identified as a limitation. This has not only hindered an integrated view of the problem, but has also hampered consistent comparisons between obtained results, ultimately resulting in a difficulty in implementing policies based on scientific evidence. Therefore, more HBM studies are needed to better characterize Portuguese exposure to the selected health-hazardous contaminants/pollutants and compare the results with total exposure levels determined in other European populations. The paucity of specific and properly validated biomarkers, as well as the lack of information on the toxicokinetics that persist for these chemicals, hinders objective risk assessments. In addition, for many chemicals, the lifetime health impacts associated with exposure remain unknown and guidance is largely missing. These limitations were in line with the main hurdles and challenges of HBM, considering the risk assessment of chemicals identified by EU and extra-EU regulators [3]. In spite of the recognized limitations, HBM makes it possible to assess trends in temporal exposure, to characterize geographical patterns of exposure, compare different population groups, and identify vulnerable subpopulations [7] to serve as the starting point for the implementation of preventive measures and assess the effectiveness of policy actions [93].
